# Age affects the diagnostic accuracy of the cancer ratio for malignant pleural effusion

**DOI:** 10.1186/s12890-023-02475-8

**Published:** 2023-06-07

**Authors:** Jin-Hong Huang, Hong Chen, Zhi-Cheng Zhang, Jie Gu, Li Yan, Meng-Ping Jiang, Wen-Qi Zheng, Zhi-De Hu, Ting-Wang Jiang

**Affiliations:** 1 Department of Pulmonary and Critical Care Medicine, the Affiliated Changshu Hospital of Nantong University, 215500 Changshu, China; 2 Department of Key Laboratory, the Affiliated Changshu Hospital of Nantong University, 215500 Changshu, China; 3 Department of Respiratory and Critical Care Medicine, the Affiliated Hospital of Inner Mongolia Medical Universit, 010050 Hohhot, China; 4 The Affiliated Hospital of Inner Mongolia Medical University, 010050 Hohhot, China; 5 Department of Laboratory Medicine, the Affiliated Hospital of Inner Mongolia Medical University, 010050 Hohhot, China

**Keywords:** Cancer ratio, Malignant pleural effusion, Sensitivity, Specificity, Diagnosis

## Abstract

**Background and objective:**

Cancer ratio (CR), which is defined as serum lactate dehydrogenase (LDH) to pleural fluid adenosine deaminase (ADA) ratio, has been reported to be a useful diagnostic marker for malignant pleural effusion (MPE). Whether its diagnostic accuracy is affected by age remains unknown. This study aimed to investigate the effects of age on the diagnostic accuracy of CR.

**Methods:**

The participants in this study were from a prospective cohort (SIMPLE cohort, n = 199) and a retrospective cohort (BUFF cohort, n = 158). All participants were patients with undiagnosed pleural effusion (PE). We used receiver operating characteristic (ROC) curves to evaluate the diagnostic accuracy of CR. The effect of age on the diagnostic accuracy of CR was investigated by adjusting the upper limit of age for participant enrolment.

**Results:**

Eighty-eight MPE patients were verified in the SIMPLE cohort, and thirty-five MPE patients were verified in the BUFF cohort. The AUCs of CR in the SIMPLE and BUFF cohorts were 0.60 (95% CI: 0.52–0.68) and 0.63 (95% CI: 0.54–0.71), respectively. In both cohorts, the AUCs of CR decreased with the advancement of age.

**Conclusion:**

Age can affect the diagnostic accuracy of CR for MPE. CR has limited diagnostic value in older patients.

**Key message:**

- Cancer ratio is a promising diagnostic marker for malignant pleural effusion.
- This study revealed that its diagnostic accuracy decreased in older patients.
- Its diagnostic accuracy is overestimated by previous studies using tuberculosis and pneumonia patients as controls.

## Introduction

Pleural effusion (PE) is a common sign in clinical practice, and its differential diagnosis is challenging for clinicians. It can be caused by various disorders, including tuberculosis, heart failure (HF), malignancy, and pneumonia [1]. PE caused by malignancy is termed malignant pleural effusion (MPE), while PE caused by non-malignant diseases is termed benign pleural effusion (BPE). Pleural fluid cytology and biopsy are the gold standards for MPE, but they have limitations [2]. Although pleural fluid cytology has the advantage of low cost, its sensitivity is between 0.40 and 0.60 [3, 4], depending on the type of cancer. Image-guided pleural biopsy or medical thoracoscopy, has high diagnostic accuracy; however, the biopsy is an invasive diagnostic tool, and operation-related complications are problematic [5]. Therefore, developing noninvasive diagnostic tools for MPE is of great value.

Biomarkers in the pleural fluid have the advantages of mini-invasiveness, low cost, and short turn-around time and thus represent valuable diagnostic tools for MPE [6]. To date, many diagnostic markers have been identified, such as neuron-specific enolase (NSE) and carcinoembryonic antigen (CEA) [7–9]. However, the diagnostic accuracy of these tumor markers, when used alone, is unsatisfactory [6]. In 2016, Vera et al. proposed that the ratio of serum lactate dehydrogenase (LDH) to the pleural fluid adenosine deaminase ratio (ADA) ratio (cancer ratio, CR) was a useful diagnostic marker for MPE [10]. Two meta-analyses revealed that CR had a high diagnostic value for MPE, with a sensitivity of 0.97 and specificity of 0.89 [11, 12]. However, the factors affecting the diagnostic accuracy of CR remain largely unknown. Previous studies have revealed that serum LDH increases with advancing age [13, 14], and the diagnostic accuracy of pleural ADA for tuberculous pleural effusion (TPE) is also affected by age [15]. Therefore, we hypothesized that age could affect the diagnostic accuracy of CR. Here, we performed a study to investigate the diagnostic accuracy of CR for MPE and the effect of age on its diagnostic accuracy. We reported our work following the Standards for Reporting of Diagnostic Accuracy Studies (STARD) guidelines [16, 17].

## Materials and methods

### Participants

This study included two cohorts, named the SIMPLE cohort and the BUFF cohort. The SIMPLE (A Study Investigating Markers in PLeural Effusion) is a prospective, double-blind diagnostic test accuracy study, and its design details have been described previously [18, 19]. In brief, patients who visited the Affiliated Hospital of Inner Mongolia Medical University (AHIMMU) with undiagnosed PE between September 2018 and July 2021 were prospectively enrolled. The exclusion criteria were as follows: (i) patients with pleural effusion within three months before admission, and the cause was clear; (ii) patients with insufficient pleural fluid specimens for the research aims; (iii) pregnant women; (iv) patients with trauma-induced PE; (v) patients who developed PE during hospitalization; and (vi) patients < 18 years old. With identical inclusion and exclusion criteria, the Affiliated Changshu Hospital of Nantong University (formerly named the Affiliated Changshu Hospital of Xuzhou Medical University) participated in this study in 2020. Patients who visited the Affiliated Changshu Hospital of Nantong University between June 2020 and July 2021 were enrolled. Because the sample size of Changshu cohort was small (n = 62), we analyzed the data of the participants in Hohhot and Changshu together, termed the SIMPLE cohort.

The second cohort is the BUFF cohort (Biomarkers for patients with Undiagnosed pleural eFFusion). BUFF is a retrospective study investigating the diagnostic value of serum or pleural biomarkers [20, 21]. The inclusion and exclusion criteria of the BUFF and SIMPLE studies were identical. We reviewed the participants’ medical records and extracted their clinical details and final diagnoses.

The ethics committees of the AHIMMU and the Affiliated Changshu Hospital of Nantong University approved the SIMPLE study (KY2018011 for Hohhot; 2020-KY-009 for Changshu), and informed consent was obtained from all participants. The BUFF study was approved by the ethics committee of the AHIMMU (NO: KY2021014). Informed consent was waived because of the retrospective design.

### Diagnosis

The diagnosis procedure was the same in the BUFF and SIMPLE cohorts. Briefly, MPE was diagnosed with pleural fluid cytology, thoracoscopy and pleural biopsy. In some patients, MPE was defined as the presence of a primary or metastatic tumor, and BPE can be excluded by their clinical characteristics and treatment response. Parapneumonic effusion (PPE) was diagnosed based on imaging, microbiology, biopsy, and response to antibiotic treatment [22]. TPE was diagnosed based on biopsy, *Mycobacterium tuberculosis (Mtb)* culture, Ziehl-Neelsen staining, and response to anti-tuberculosis therapy. HF was diagnosed based on the clinical findings, imaging features (decreased left ventricular ejection fraction, enlarged heart shadow), laboratory tests (e.g., serum natriuretic peptides), transudate and response to diuretics.

### Statistical analysis

We used the Kolmogorov-Smirnov method to test the distribution of continuous variables, including serum and pleural fluid biochemistries. The independent Student’s t-test was used to compare the means of the continuous variables with a normal distribution (e.g., serum protein), and the Mann-Whitney U test was used to compare the continuous variables with skewed distribution (e.g., pleural LDH, ADA, protein; CR; serum LDH). The Chi-square test was used to compare categorical variables (e.g., sex). The diagnostic accuracy of CR (serum LDH to pleural ADA ratio) for MPE was assessed with receiver operating characteristic (ROC) curves. To determine the effect of age on the diagnostic accuracy of CR for MPE, we resampled patients by adjusting the upper limit of age for the patient selection. For example, we set the upper limit of age to 55 years and only analyzed the diagnostic accuracy of CR in patients under 55 years. The details of this method have been reported in our previous study [20]. All analyses were performed using R software (version. 4.3.1). A *p*-value < 0.05 was defined as statistically significant.

## Results

### Characteristics of the participants

**Fig. 1:**
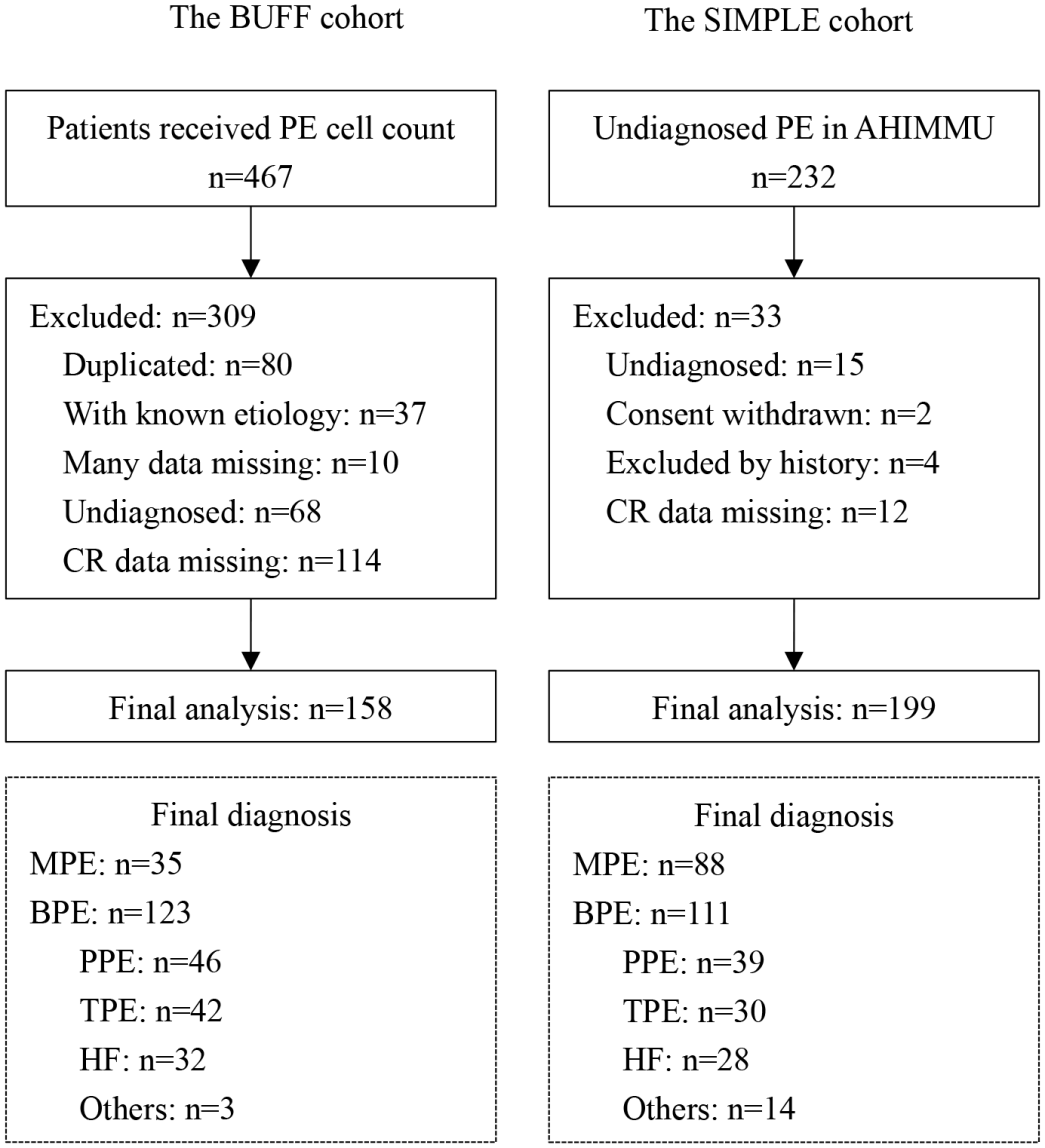
Flowchart of the participant selection process. TPE, tuberculous pleural effusion; PPE, parapneumonic pleural effusion; HF, heart failure; MPE, malignant pleural effusion; BPE, benign pleural effusion; CR, cancer ratio; PE, pleural effusion.

Figure 1 is a flowchart of the participant selection procedure. A total of 357 participants with undiagnosed PE were enrolled in this study. In the BUFF cohort, 158 participants (35 MPEs and 123 BPEs) were enrolled. In the SIMPLE cohort, 199 participants (88 MPEs and 111 BPEs) were enrolled. The clinical characteristics of the participants are listed in Table 1. The median (quartile) ages (in years) of TPE, MPE and PPE in the SIMPLE cohort were 73 (65–80), 73 (67–79) and 69 (60–76), respectively. In the BUFF cohort, the median (quartile) ages (in years) of TPE, MPE and PPE were 67 (44–76), 64 (57–72) and 65 (58–73), respectively. Similar to previous studies [10, 12, 23–26], increased CR was observed in the MPE patients.

**Table 1:** Characteristics of the participants

Characteristics	BUFF cohort (n = 158)			SIMPLE cohort (n = 199)						
	BPE (n = 123)	MPE (n = 35)	p	BPE (n = 111)		MPE (n = 88)		p		
Sex n (%)			0.381					0.277		
Female	34 (28)	13 (37)		36 (32)		36 (41)				
Male	89 (72)	22 (63)		75 (68)		52 (59)				
Age, years	69 (58, 78)	64 (57, 72)	0.129	72 (64, 80)		73 (67, 79)		0.515		
Pleural fluid biochemistry										
WBC, 10^6^/mm3	993 (445, 2191)	1652 (656, 2338)	0.289	818 (361, 2115)	922 (670, 1522)		0.496		0.496	
Glucose, mmol/L	5.5 (3.8, 6.7)	5.4 (3.9, 6.8)	0.708	5.8 (4.6, 7.1)		6.1 (5.1, 6.7)		0.650		
LDH, U/L	238 (115, 735)	306 (173, 620)	0.308	195 (105, 471)		291 (187, 462)		0.011		
ADA, U/L	15 (7, 40)	10 (7, 12)	0.017	13 (5, 35)		9 (6, 13)		0.067		
Protein, g/L	24 (17, 38)	25 (19, 36)	0.439	34 (19, 44)		39 (33, 43)		0.012		
Serum biochemistry										
Protein, g/L	64 ± 8	64 ± 7	0.677	61 ± 9		64 ± 8		0.025		
LDH, U/L	213 (177, 257)	194 (176, 248)	0.515	206 (172, 255)		217 (179, 262)		0.164		
Cancer ratio	12.2 (5.4, 33.7)	21.8 (14.1, 32.0)	0.024	19.0 (6.4, 40.2)		24.8 (16.3, 41.0)		0.018		

Normally distributed continuous data were expressed as the mean ± standard deviation (SD), and skewed distributed continuous data were expressed as the median and quartile. Categorical data were expressed as absolute numbers and percentages. WBC, white blood cell; ADA, adenosine deaminase; LDH, lactate dehydrogenase; BPE, benign pleural effusion; MPE, malignant pleural effusion.

### Diagnostic accuracy of CR for MPE

**Fig. 2:**
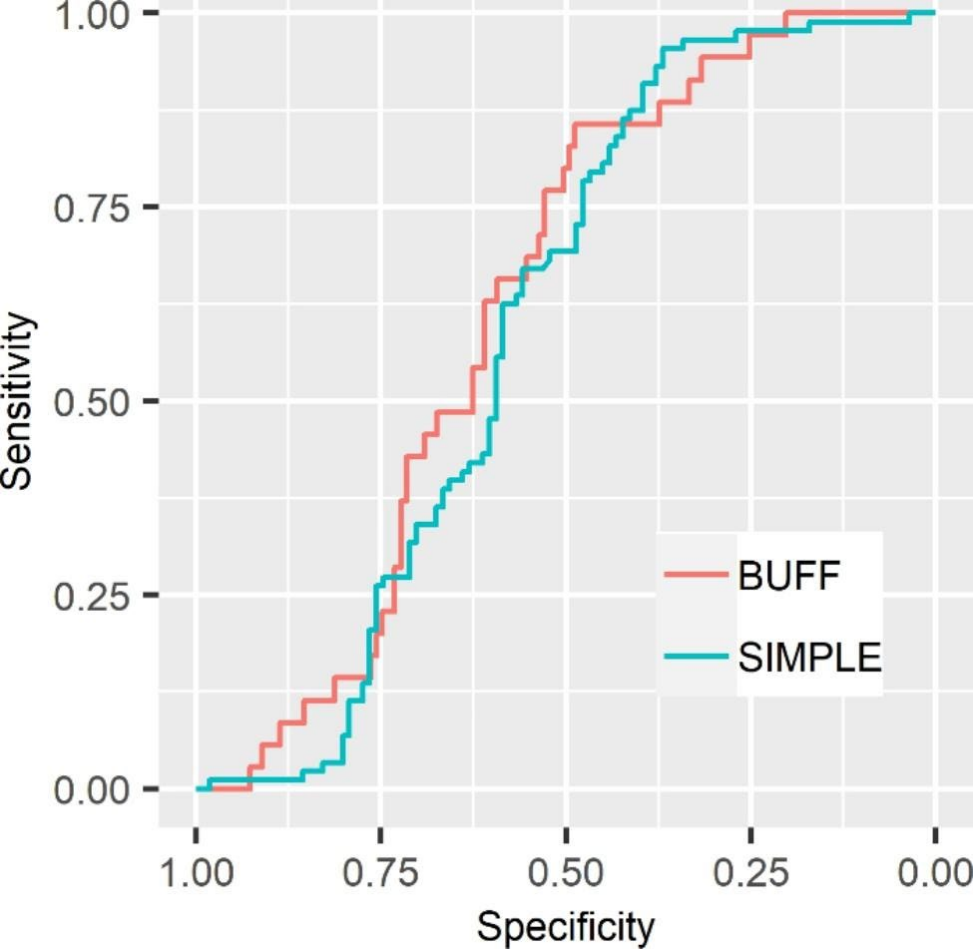
ROC curves for CR

Figure 2 shows the ROC curves of the CR for MPE. The AUCs (95% CI) of the CR in the BUFF and SIMPLE cohorts were 0.63 (0.54–0.71) and 0.60 (0.52–0.68), respectively. Table 2 summarizes the sensitivity and specificity of CR. Because data-driven threshold selection can overestimate the diagnostic accuracy of a test [27], we prespecified the threshold of CR at 20, which is adopted by a previous study [10].

**Table 2:** Sensitivity and specificity of CR for MPE

Diagnostic metrics	BUFF cohort		SIMPLE cohort	
	All	MPE/TPE/PPE	ALL	MPE/TPE/PPE
Threshold	20	20	20	20
AUC (95% CI)	0.63 (0.54–0.71)	0.75 (0.66–0.83)	0.60 (0.52–0.68)	0.77 (0.68–0.85)
Sensitivity (95% CI)	0.57 (0.40–0.74)	0.57 (0.40–0.74)	0.67 (0.57–0.76)	0.67 (0.57–0.76)
Specificity (95% CI)	0.61 (0.53–0.69)	0.76 (0.67–0.85)	0.54 (0.44–0.63)	0.72 (0.61–0.83)

AUC, the area under the curve; CI, confidence interval; MPE, malignant pleural effusion; TPE, tuberculous pleural effusion; PPE, parapneumonic pleural effusion

### Effects of age and components of non-MPE on the diagnostic accuracy of CR

We searched the PubMed database and found that several studies have investigated the diagnostic accuracy of CR for MPE. The characteristics of these studies are summarized in Table 3. The AUCs in the previous studies were higher than those in our cohorts. We noted that the ages of the participants in previous studies were younger than those in our cohort, and the components of BPE in the previous studies were primarily TPE and PPE. Therefore, we hypothesized that the diagnostic accuracy of CR is affected by the age of the participants and the components of BPE. As shown in Table 2, when we only included MPE, PPE or TPE in the final analysis, the AUCs of the CR in both the BUFF and SIMPLE cohorts increased.

**Table 3:** Characteristics of the studies investigating the diagnostic accuracy of CR for MPE

First author	Year	Median or mean age, years			BPE components	AUC	Threshold	Sensitivity	Specificity
		All	MPE	BPE					
Verma [10]	2016	NR	NR	NR	TPE, PPE	0.81	20.00	0.95	0.85
Verma [28]	2016	65	69	56	TPE	0.81	20.00	0.98	0.94
Elmahalawy [29]	2017	67	68	~ 66	TPE, PPE	1.00	5.03	1.00	0.87
Zhang [30]	2017	NR	64	~ 62	TPE, PPE	0.84	10.60	0.94	0.73
Korczyński [25]	2018	NR	69	~ 55	TPE, PPE	0.83	16.40	0.95	0.69
Gayaf [26]	2021	61	NR	NR	TPE, PPE	0.73	14.25	0.84	0.53
Zhang [12]	2021	NR	61	54	TPE, PPE, HPE, AP, CT	0.86	14.97	0.91	0.67
Ren [23]	2021	NR	72	31	TPE	0.85	19.20	0.81	0.87
Zhang [24]	2022	NR	69	60	TPE, PPE	0.79	20.48	0.83	0.70
Zhou [31]	2023	56	64	39	TPE	0.86	21.24	0.80	0.80
Gao [32]	2023	NR	69	64	TPE, PPE	0.88	12.50	0.95	0.67
This study, BUFF	2023	68	64	69	TPE, PPE, HF, others	0.63	20.00	0.57	0.61
This study, SIMPLE	2023	73	72	73	TPE, PPE, HF, others	0.60	20.00	0.67	0.54

BPE, benign pleural effusion; MPE, malignant pleural effusion; TPE, tuberculous pleural effusion; PPE, parapneumonic pleural effusion; HPE, hepatic pleural effusion; AP, acute pancreatitis; CT, chylothorax; HF, heart failure; NR, not reported

**Fig. 3:**
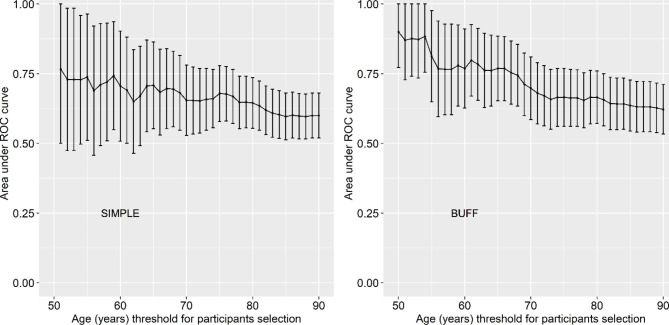
Effect of participants’ age on the area under the curve of cancer ratio

Figure 3 depicts the effect of the participant’s age on the AUC of CR. In both the BUFF cohort and SIMPLE cohort, the AUCs of CR decreased as the upper limit of the age for enrolment increased. For example, in the BUFF cohort, when we included the participants under 50 years in the analysis, the AUC of CR was 0.90, but when we included the patients under 90 years, the AUC of CR was only 0.62.

## Discussion

In this study, we validated the diagnostic accuracy of CR for MPE. With two cohorts, we found that the diagnostic accuracy of CR for MPE was limited because its AUC was < 0.70. The accuracy of CR was affected by the participant’s age, and it was decreased in old patients. In addition, the component of BPE may be another factor affecting the diagnostic accuracy of CR.

Compared with previous studies [10, 23–25, 28, 30], our study has its strengths. The first strength is that we used two cohorts to investigate the diagnostic accuracy of CR. The results in these two cohorts were similar, indicating that the findings of our study are reliable. Second, we found that the diagnostic accuracy of CR decreased with increasing age, indicating that CR may not be a useful diagnostic marker for MPE in older patients. Third, we found that the diagnostic accuracy of CR was affected by the disease spectrum of BPE patients. A large portion of the previous studies used TPE and PPE patients as controls and found that the diagnostic accuracy of CR was promising **(**Table 3**)**. To date, more than 50 causes of PE have been recognized [33], and patient selection bias may occur when only including TPE, PPE and MPE patients. One strength of our study is that we enrolled patients with prespecified inclusion and exclusion criteria, which is termed a one-gate design [34, 35]. In addition to PPE and TPE, other types of PE patients (e.g., HF, pulmonary embolism, connective tissue disease) were also enrolled in our study. Therefore, our study cohort is more representative than those in previous studies.

We found that age could affect the diagnostic accuracy of CR. This finding is biologically plausible. The definition of CR is the ratio of serum LDH to pleural ADA. These two biomarkers are affected by age, as reported by previous studies. Specifically, pleural ADA was inversely correlated with age in patients with pleural effusion [36, 37], and its diagnostic accuracy for TPE was decreased in old patients [15]. In addition, serum LDH increases with the advancement of age [14, 38]. Therefore, elderly patients have higher CR than young patients, and age should be considered when interpreting the diagnostic value of CR for MPE [23]. It is widely accepted that PPE patients have increased serum LDH, and TPE patients have increased pleural ADA. Therefore, it is natural that the diagnostic accuracy of CR improves when researchers include only TPE and PPE as BPE.

Someone may argue that the direct factor that affects the diagnostic accuracy of CR may not be “age” but “the percentage of TPE among BPE.“ As shown in Table 3, the age of included patients in each study becomes younger when the study includes only TPE as the control. Indeed, TPE patients in previous studies aged around 40 years old [10, 23–25, 28–30]. However, the median age of TPE patients in our study is around 70 years old, which is obviously higher than that in previous studies. Therefore, it seems that the limit of age for participant enrollment (Fig. 3) does not affect the percentage of TPE among BPE.

Although CR is a low-cost diagnostic marker for MPE, our study revealed that its AUC is approximately 0.60, indicating that its diagnostic accuracy is unsatisfactory. However, in the BUFF study, we observed that the AUC was approximately 0.90 in patients aged < 50 years. Therefore, we conclude that CR may be a useful diagnostic marker for MPE in non-elderly patients. However, because the sensitivity and specificity of CR are not 1.00, the results of CR should be interpreted along with the patient’s clinical signs, symptoms and other laboratory results.

Our study has some limitations. The first limitation is the retrospective design of the BUFF cohort, which may affect the representativeness of the participants. The second limitation is that a large portion of the participants are from the AHIMMU, and previous studies have revealed that the causes of PE vary across different countries or regions. Notably, the mean age of participants in our cohorts was higher than that in previous studies. The final diagnoses of the participants and their prevalence may affect the diagnostic accuracy [34, 39]. Therefore, caution should be taken when extending our findings to other areas.

Taken together, our study found that the diagnostic accuracy of CR for MPE is affected by age and the disease spectrum of BPE. Its diagnostic accuracy decreases in old patients. Therefore, age and disease spectrum should be considered when interpreting the results of CR.

## Data Availability

The datasets generated and/or analysed during the current study are not publicly available due ethical restrictions but are available from the corresponding author on reasonable request.
